# Analysis of the shorter drug survival times for Janus kinase inhibitors and interleukin-17 inhibitors compared with tumor necrosis factor inhibitors in a real-world cohort of axial spondyloarthritis patients - a retrospective analysis from the RHADAR network

**DOI:** 10.1007/s00296-024-05671-9

**Published:** 2024-08-13

**Authors:** Patrick-Pascal Strunz, Matthias Englbrecht, Linus Maximilian Risser, Torsten Witte, Matthias Froehlich, Marc Schmalzing, Michael Gernert, Astrid Schmieder, Peter Bartz-Bazzanella, Cay von der Decken, Kirsten Karberg, Georg Gauler, Patrick Wurth, Susanna Späthling-Mestekemper, Christoph Kuhn, Wolfgang Vorbrüggen, Johannes Heck, Martin Welcker, Stefan Kleinert

**Affiliations:** 1 Department of Medicine II, Rheumatology/Clinical Immunology, University Hospital of Wuerzburg, Oberdürrbacher Straße 6, 97080 Wuerzburg, Germany; 2 Freelance Healthcare Data Scientist, Greven, Germany; 3 Department of Rheumatology and Immunology, Medical School Hannover, Hannover, Germany; 4 Clinic for Dermatology, Venereology and Allergology, University Hospital Wuerzburg, Wuerzburg, Germany; 5 Klinik für Internistische Rheumatologie, Rhein-Maas-Klinikum, Würselen, Germany; 6 Medizinisches Versorgungszentrum, Stolberg, Germany; 7 Rheumatologisches Versorgungszentrum Steglitz, Berlin, Germany; 8 Rheumatology Practice, Osnabrück, Germany; 9 Rheumapraxis München, München, Germany; 10 Praxis für Rheumatologie, Karlsruhe, Germany; 11 Verein zur Förderung der Rheumatologie e.V., Würselen, Germany; 12 Hannover Medical School, Institute for Clinical Pharmacology, Hannover, Germany; 13 Medizinisches Versorgungszentrum für Rheumatologie Dr. M. Welcker GmbH, Planegg, Germany; 14 Praxisgemeinschaft Rheumatologie-Nephrologie, Erlangen, Germany

**Keywords:** Bechterew´s disease, Ankylosing spondylitis, Drug persistence, Kaplan-meier analysis, Upadacitinib, Tofacitinib, Secukinumab, Ixekizumab, Adalimumab

## Abstract

In recent years Janus kinase inhibitors (JAKi) have joined tumor necrosis factor inhibitors (TNFi) and interleukin (IL)-17 inhibitors (IL-17i) as approved disease modifying anti-rheumatic drugs (DMARD) for moderate to severe forms of axial spondyloarthritis (axSpA). Drug survival in axSpA patients has not been well studied in a real-world outpatient scenario since the approval of JAKi. We aimed to analyze the three drug classes based on modes of actions (MoA) for their persistence rates among German axSpA outpatients. A retrospective analysis of the RHADAR database for axSpA patients with a new initiation of TNFi, IL-17i, or JAKi treatment between January 2015 and October 2023 was conducted. Analyses included Kaplan-Meier curves and adjusted Cox regressions for drug discontinuation. 1222 new biological DMARD (TNFi [*n* = 954], IL-17i [*n* = 190]) or JAKi (*n* = 78) treatments were reported. The median drug survival was 31 months for TNFi, 25 for IL-17i, and 18 for JAKi. The corresponding 2-year drug survival rate was 79.6%, 72.6%, and 62.8% for TNFi, IL-17i, and JAKi, respectively. The probability for discontinuation for JAKi was significantly higher compared with TNFi (HR 1.91 [95% CI 1.22–2.99]) as well as for IL-17i compared with TNFi (HR 1.43 [95% CI 1.02–2.01]), possibly related to more frequent use of TNFis as first-line therapy. IL-17i and JAKi discontinuation probabilities were similar. Primary non-response was the reason for drug discontinuation in most cases across all MoA. TNFi treatment might persist longer than JAKi and IL-17i in German axSpA outpatients, possibly related to more severe or refractory disease in patients with JAKi-treated or IL-17i-treated axSpA.

**Supplementary Information:**

The online version contains supplementary material available at 10.1007/s00296-024-05671-9.

## Introduction

In addition to disease activity and treatment response measures, treatment persistence is another crucial factor when considering and comparing treatment efficacy in axial spondyloarthritis (axSpA), a rheumatic disease with the focus on the axial skeleton [1–3]. In this context, the term “drug survival” was introduced to depict the likelihood of patients staying on a specific medication [2, 3]. Drug survival is thus used as an approximation of the efficacy and safety of a drug [2, 3].

Studying drug survival in a real-world scenario in outpatient care distinct from specialized study centers is difficult due to the challenge of collecting suitable and adequate data [4]. New digital tools and smart databases can help with collecting and evaluating patients’ data for study purposes, especially when the disease is seldom like axSpA. The RHADAR network was founded to aggregate real-world data on patients with rheumatic diseases [4]. It currently consists of seven rheumatic practices and one outpatient department of a hospital in Germany, specialized in rheumatology, that supply pseudonymized data for aggregation into the RHADAR joint database [4].

In axSpA, treatment options with disease-modifying antirheumatic drugs (DMARD) have evolved over the last decade. Three drug classes with different modes of action (MoA) have been approved for moderate to severe axSpA in Europe and the USA as of February 2024, specifically tumor necrosis factor alpha inhibitors (TNFi), interleukin (IL)-17 inhibitors (IL-17i), and Janus kinase inhibitors (JAKi) [5, 6]. While drug survival of TNFi and IL-17i has been studied intensively over the last decade, sufficient data for JAKi, the drug class most recently approved, are still not widely available [3, 7–11]. Similarly, the impact of orally available agents such as JAKi on real-world persistence with bDMARDs delivered by more invasive methods still needs to be further studied in axSpA.

In this analysis, we aimed to assess the drug survival of JAKi compared with bDMARDs (TNFi and IL-17i) among German outpatients treated for axSpA under real-world conditions.

## Methods

### Study design

Using the RHADAR database, a retrospective analysis was performed for axSpA patients who initiated treatment with a TNFi, IL-17i, or JAKi between January 15th 2015 and October 17th 2023. All approved IL-17i (ixekizumab, secukinumab), TNFi (adalimumab, certolizumab, etanercept, golimumab, and infliximab), and JAKi (tofacitinib and upadacitinib) were clustered by MoA and included in the analysis. Bimekizumab was not included in the analysis due to the short period since its regulatory approval for axSpA (June 7th 2023) [12]. Similar analyses on drug survival of JAKi and different bDMARDS among German psoriatic arthritis outpatients from the RHADAR database using the same methodology have already been published elsewhere [13].

### Patients

Patients included in the analyses had a diagnosis of axSpA as indicated by International Classification of Disease-10 (ICD-10) codes (German 2023 version) and had previously given written informed consent to be included in the RHADAR database and to allow the use of their pseudonymized data for analyses. All patients were treated in outpatient departments in Germany (i.e. seven rheumatologic practices and one outpatient department of a rheumatologic hospital). The treating rheumatologists assessed the relevant demographic factors and axSpA activity parameters, including Bath Ankylosing Spondylitis Disease Activity Index (BASDAI, ranging from 0 to 10), Bath Ankylosing Spondylitis Functional Index (BASFI, range 0 to 10), Ankylosing Spondylitis Disease Activity Score (ASDAS with a range from 0 to > 3.5), and laboratory parameters, during routine clinical care [14].

### Study size

A sample size calculation was not performed due to the retrospective character of the study. Accordingly, the number of database entries determined the number of axSpA patients available for the analysis.

### Ethical approval

The patients gave written informed consent for pseudonymized Inclusion in RHADAR database and for analysis of their data. The ethics committee of the Medical Faculty of the University of Wuerzburg (DE/EKBY13) stated that the study represents a retrospective data analysis of pseudonymzied data assessed in clinical routine care. Therefore, ethical approval by German law is not required when the publication of data is performed in anonymous form (Date February 19th, 2024, No 20240122 03).

### Statistical analysis

We performed a Kaplan-Meier (KM) analysis for comparison of the drug persistence of each MoA over 24 months, similar to our previous work concerning PsA patients [13]. An event was defined as discontinuation of a bDMARD or JAKi therapy. Demographic parameters, axSpA specific disease parameters, combined medication, and comorbidities were used to characterize the overall and MoA-specific patient populations. For sample characterization, the following descriptive measures were used: absolute and relative frequencies, means with standard deviation (SD), 95% confidence intervals (CI) of the mean, and medians with interquartile range (25th and 75th percentiles). If not stated otherwise, mean (SD) values are reported.

To assess the influence of MoA on drug discontinuation risk over time, we conducted Cox regression analysis with the MoA exhibiting the longest drug persistence serving as reference. Drug survival rates at month x were calculated by the formula (n [at risk at month x) + n [cumulative censored at month x]) ÷ n [at risk at month 0]. The corresponding model was adjusted for age, sex, and disease duration as potential confounders. Hazard ratios (HRs) with corresponding 95% CIs were reported for each MoA as well as each covariate (age, gender, disease duration). Statistical significance was set at *p* ≤ 0.05. Statistical analyses were performed using R software (version 4.4.0) and RStudio (version 2023.12.1 + 402) [15, 16]. For dichotomous variable distribution analysis, 95% CIs, odds ratios (OR), and chi-square tests were utilized. Prism Version 5 was utilized for these analyses. Missing values were not imputed to preserve the original data integrity. To evaluate treatment response for discontinued therapies, BASDAI and ASDAS changes from start of the discontinued therapy to the start of the subsequent prescription were calculated.

## Results

### Study population

A new prescription for a bDMARD or JAKi therapy was reported for 1222 axSpA cases with additional patient characteristics (668 males [54.7%] and 554 females [45.3%]) from the RHADAR database. Patient characteristics obtained at the start of the therapy are detailed in Table 1. A mean (SD) age of 47.0 years (13.4 years) was found. The average BASDAI was 4.3 (2.3) and the mean ASDAS was 2.5 (0.9).

**Table 1:** Patient characteristics at treatment initiation (*N* = 1222)

	Valid (*n*)	Valid (%)	Mean	95%CI (lower)	95%CI (upper)	SD	SEM	Median	25% Quantile	75% Quantile
Gender (male)	668	54.7	NA	NA	NA	NA	NA	NA	NA	NA
Gender (female)	554	45.3	NA	NA	NA	NA	NA	NA	NA	NA
Age	1222	100.0	47.0	46.2	47.7	13.4	0.4	47.0	37.0	57.0
Disease duration (years)	1047	85.7	12.6	11.9	13.3	11.3	0.3	9.0	4.0	19.0
BASDAI	807	66.0	4.3	4.2	4.5	2.3	0.1	4.4	2.5	6.0
BASFI	595	48.7	3.6	3.4	3.8	2.7	0.1	3.2	1.3	5.7
ASDAS	421	34.5	2.5	2.4	2.6	0.9	0.0	2.5	1.8	3.1
ESR (mm/h)	637	52.1	18.6	17.3	20.0	17.5	0.7	12.0	8.0	24.0
CRP (mg/dl)	776	63.5	0.7	0.6	0.9	2.0	0.1	0.3	0.1	0.6
Pain (0-100)	368	30.1	41.7	38.8	44.5	27.9	1.5	41.0	16.0	64.2
Morning stiffness (min)	342	28.0	70.4	51.4	89.3	178.6	9.7	30.0	16.2	60.0

### Treatment persistence

**Fig. 1:**
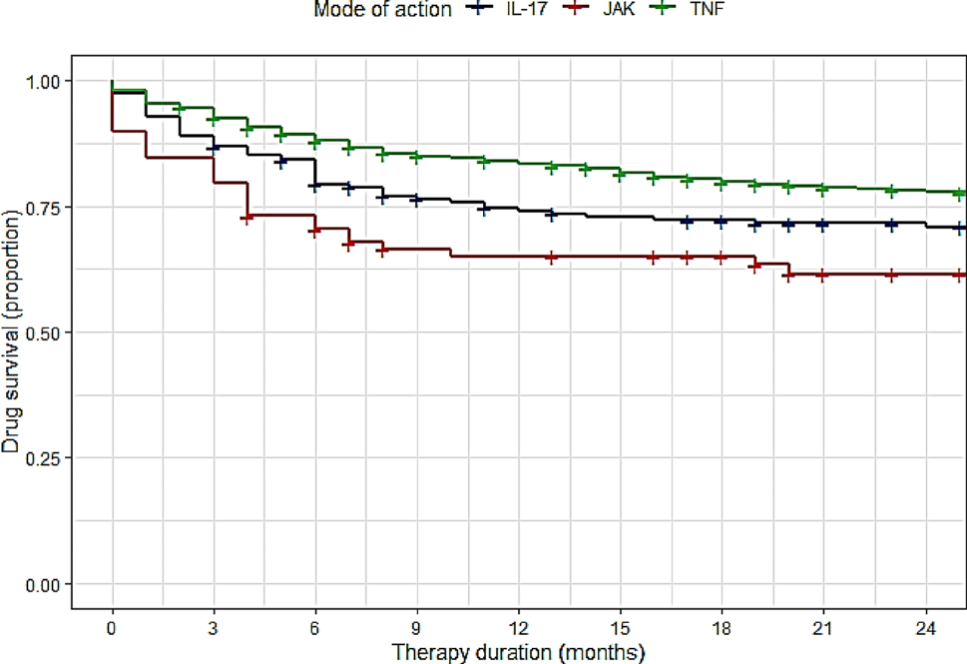
Drug persistence in axSpA patients

During the analyzed period, 1222 new treatments with bDMARDs (TNFi [954 cases], IL-17i [190 cases]) or JAKi (78 cases) were reported. The median drug survival was 31 months for TNFi, 25 months for IL-17i, and 18 months for JAKi. Figure 1 shows the KM diagram for drug survival of the different MoA. Most discontinuations occurred within the first 12 months of treatment initiation. After this initial period, discontinuation rates decreased, leading to fairly constant persistence rates for a given drug class from 1 to 2 years (Table 2). The corresponding 1-year drug survival probability was 84.4% for TNFi, followed by 75.3% for IL-17i and 65.4% for JAKi. A 2-year drug survival rate of 79.6% was found for TNFi compared with 72.6% for IL-17i and 62.8% for JAKi (Table 2).

**Table 2:** Risk table

Mode of action	Month	At risk (*n*)	At risk (%)	Cumulative events (*n*)	Cumulative censored (*n*)	Strata size (*n*)	Drug survival propability
IL-17i	0	190	100	5	0	190	100.0%
IL-17i	12	122	64	48	21	190	75.3%﻿
IL-17i	24	99	52	53	39	190	﻿72.6%
JAKi	0	78	100	8	0	78	﻿100.0%
JAKi	12	46	59	27	5	78	﻿65.4%
JAKi	24	26	33	29	23	78	﻿62.8%
TNFi	0	954	100	19	0	954	﻿100.0%
TNFi	12	702	74	155	103	954	﻿84.4%
TNFi	24	559	59	198	200	954	﻿79.6%

Cox regression with TNFi serving as the reference drug class indicated JAKi (HR 1.91 [95% CI 1.22–2.99]) and IL-17i (HR 1.43 [95% CI 1.02–2.01]) to have a significantly higher probability of drug discontinuation compared to TNFi. No difference was observed between JAKi and IL-17i in a supplemental Cox regression with IL-17i serving as the reference drug class (HR 1.33 [95% CI 0.79–2.24]).

Female gender (with male gender as reference) (HR = 1.97 [95% CI 1.50–2.59 ]) and shorter disease duration (HR = 0.98 [95% CI 0.97–0.99]) were found as independent covariates affecting drug survival in Cox regression analyses.

### Changes in disease activity during treatment

Due to the structure of the RHADAR database, a direct data query for reasons of drug discontinuation was not possible. To gain insights into whether adverse events or loss of effectiveness were the main cause for drug discontinuation, we analyzed the available changes regarding BASDAI and ASDAS during treatment for each MoA (Table 3). Corresponding change scores ranged from − 0.5 to − 0.7 for BASDAI and were equal to − 0.1 for ASDAS for all drug classes included. We only found mild negative delta values and no differences in the mean delta BASDAI or mean delta ASDAS between the three MoA, indicating that the main cause for treatment change was primary loss of efficacy and not adverse events. No difference was seen between the three MoA.

**Table 3:** BASDAI and ASDAS changes from start of a new therapy until discontinuation

Assessment	TNFi (*n* = 198)		IL-17i (*n* = 53)		JAKi (*n* = 29)	
	*n* (%)	Mean (95% CI)	*n* (%)	Mean (95% CI)	*n* (%)	Mean (95% CI)
BASDAI	106 (53.5)	− 0.5 (0.2 − 0.8)	30 (56.6)	− 0.7 (0.2 − 1.1)	18 (62.1)	-0.6 (0.1–1.2)
ASDAS	48 (24.2)	-0.1 (-0.1-0.3)	13 (24.5)	− 0.1 (-0.1 -0.3)	10 (34.5)	-0.1 (0.0–0.3)

### Subgroup analysis stratified by drug classes

Potential effects of varying group characteristics were analyzed to reveal potential impacts on drug survival (see Table 4 for an overview of the characteristics by drug class and supplemental section S1 for additional details).

**Table 4:** Patient characteristics stratified by treatment MoA

Characteristic	TNFi (*n* = 954 )		IL-17i (*n* = 190)		JAKi (*n* = 78)	
	*n* (%)	Mean (95% CI)	*n* (%)	Mean (95% CI)	*n* (%)	Mean (95% CI)
Age	954 (100)	46.7 (45.8–47.6)	190 (100)	47.0 (45.3–48.8)	78 (100)	50.0 (47.0–53.0)
Gender (male)	528 (55.3)	-	105 (55.3)	-	35 (44.9)	-
Gender (female)	426 (44.7)	-	85 (44.7)	-	43 (55.1)	-
Disease duration (years)	823 (86.3)	12.6 (11.8–13.4)	160 (84.2)	12.7 (10.9–14.4)	64 (82.1)	12.7 (10.1–15.2)
BASDAI	624(65.4)	4.1 (3.9–4.2)	127 (66.8)	5.3 (4.9–5.6)	56 (71.8)	5.2 (4.6–5.7)
BASFI	461 (48.3)	3.3 (3.1–3.6)	91 (47.9)	4.6 (4.1–5.2)	43 (55.1)	4.7 (3.9–5.6)
ASDAS	311 (32.6)	2.4 (2.3–2.5)	70 (36.8)	2.8 (2.6–3.0)	40 (51.3)	3.0 (2.7–3.3)
ESR (mm/h)	487 (51.0)	18.6 (17.0–20.1)	102 (53.7)	18.3 (15.1–21.6)	48 (61.5)	19.9 (14.9–24.9)
CRP (mg/dl)	589 (61.7)	0.6 (0.5–0.8)	130 (68.4)	1.1 (0.5–1.8)	57 (73.1)	0.8 (0.4–1.2)
Pain (0-100)	273 (28.6)	37.0 (33.8–40.2)	64 (33.7)	54.8 (48.7–61.0)	31 (39.7)	55.3 (45.4–65.3)

Patients in the TNFi subgroup had slightly lower disease activity parameter (BASDAI, ASDAS) compared with IL-17i- and JAKi-treated patients (BASDAI of 4.1 [TNFi] vs. 5.2 [JAKi] vs. 5.3 [IL-17i], ASDAS of 2.4 [TNFi] vs. 3.0 [JAKi] vs. 2.8 [IL-17i]) when considering the 95% confidence intervals. No difference was found between the JAKi and IL-17i subgroups. TNFi-treated patients had an average of 0.5 previous bDMARD/tsDMARD treatments, IL-17i-treated patients received an average of 1.1 prior treatments, and JAKi-treated patients had an average of 1.9 previous therapies.

### Simultaneous conventional synthetic DMARD therapies and relevant comorbidities with influence on drug survival

Combination with a conventional synthetic DMARD (csDMARD) and comorbidities might affect drug survival [17–21]. Firstly, we therefore analyzed the RHADAR database to assess whether the bDMARD or JAKi therapies were administered as monotherapy or in combination with a csDMARD. There were no significant differences in the frequencies of concomitant treatment among the three drug classes (TNFi 11.0%, JAKi 16.7%, IL-17i 8.9%) (see also Supplemental section S2). Methotrexate was the most common csDMARD used in concomitant therapy (IL-17i 8.4%, JAKi 15.4%, and TNFi 10.3%). Sulfasalazine was seldom used in combined therapy (IL-17i 0.5%, JAKi 1.3%, TNFi 0.6%) and hydroxychloroquine only in one TNFi patient (0.1%).

Secondly, we analyzed the drug classes for their distribution of reported comorbidities based on the database entries (Table 5). While osteoarthritis rates were similar among the three subgroups, depression was less common in the TNFi (2.7%) compared with the IL-17i (6.3%) and JAKi (5.1%) groups. Coronary heart disease as well as overall cardiovascular risk factors (except for obesity) were similarly distributed. Smoking and diabetes mellitus were not reported in the JAKi group. Regarding axSpA-specific comorbidities, the highest rate of uveitis was found in the JAKi group (16.7% vs. 5.3% [IL-17i] vs. 4.4% [TNFi]) and Crohn’s disease/ inflammatory bowel diseases were only reported in the TNFi group (2.3%).

**Table 5:** Comorbidities by drug class

Comorbidity^a^	TNFi (*n* = 954)	IL-17i (*n* = 190)	JAKi (*n* = 78)
Osteoarthritis	169 (17.7%)	45 (23.7%)	18 (23.1%)
Uveitis	42 (4.4%)	10 (5.3%)	13 (16.7)
Depression	26 (2.7%)	12 (6.3%)	4 (5.1%)
Chronic inflammatory bowel disease	22 (2.3%)	0	0
Coronary heart disease	39 (4.1%)	11 (5.8%)	4 (5.1%)
Obesity	35 (3.7%)	11 (5.8%)	5 (6.4%)
Cardiovascular risk factors	340 (35.6%)	78 (41.1%)	24 (30.8%)
Missing	110 (11.5%)	17 (9.0%)	1 (1.3%)
No comorbidity	21 (2.2%)	2 (1.1%)	1 (1.3%)

Data are reported as n (%)

^a^Based on ICD-10 codes: osteoarthritis, M15-M19; depression, F32; coronary heart disease, I25; obesity, E66; cardiovascular risk factors, I10, E78, E11-E14; F17; uveitis, H20; chronic inflammatory bowel disease/ Crohn´s disease K50

## Discussion

In this study, we provide new data on the drug survival of different drug classes (TNFi, IL-17i, and JAKi) among German axSpA outpatients treated during routine care since approval of JAKi. Most new treatments were initiated with a TNFi, followed by IL-17i and JAKi. The highest drug survival probability was found for TNFi, followed by IL-17i. The lowest rates of persistence were seen in JAKi-treated patients. The most common time period for treatment discontinuation was during the first 12 months after treatment initiation due to lack of effectiveness. Discontinuation subsequently declined, and drug persistence rates remained fairly constant between 12 and 24 months. Using Cox regression models, we found significantly higher adjusted HRs for JAKi and IL-17i drug discontinuation than for TNFi. The risk for discontinuation of IL-17i did not differ significantly from JAKi.

Comparing our study population with the reported German average axSpA population, our patients were slightly younger (mean of 47.0 years [13.4 years]) than those described in two claims database studies (61.2 and 56.5 years), but the male to female ratio was quite similar [22, 23]. Neither database analyses reported disease parameters such as BASDAI [22, 23]. Two other smaller studies of German axSpA cohorts reported generally similar characteristics to our cohort for age (55.9 years/56.1 years) and gender distribution (46.4% female proportion for both) as well as for BASDAI (4.5 for both) [24, 25]. Therefore, we conclude that our study population is representative of the German axSpA population and that our findings are likely to be transferable to other axSpA patients in Germany.

A 2020 meta-analysis of drug survival for all TNFi in ankylosing spondylitis (AS) revealed drug survival rates of 76.0% at one year and around 60–70% at year 2 which is slightly lower than our reported 1-year and 2-year drug survival rate [7]. However, in the study mentioned, the drug survival rates for TNFi showed high variability ranging from 30 to 100% (supplementary file to [7]). Accordingly, interpreting this discrepancy between our data and these reported metadata is challenging. One explanation might be the dualism in the German healthcare system: only treated outpatients were included in the RHADAR database. Disease severity in these patients might be lower than those with more refractory or difficult-to-manage axSpA, who are more likely to be treated in specialized clinical departments and not in rheumatologic practices. Additionally, the TNFi patients in our cohort had a mean of 0.5 previous treatments and, therefore, were often bDMARD naïve, with the administered TNFi being their first-line treatment. Importantly, first-line treatments were found to have higher retention rates than second- or third-line treatments [26]. In line with this observation, the aforementioned meta-analysis found drug retention rates of around 82% for etanercept and of around 80% for adalimumab in bDMARD-naïve patients at year 1, which is similar to our reported rate [7]. A further explanation could be that the broader availability of magnetic resonance imaging and improved diagnostic procedures in Germany over recent years may help to better distinguish flares and disease activity from back pain and degenerative disorders, leading to fewer treatment switches due to perceived treatment failure.

Our observed retention rates for IL-17i were in in line with other reported real-world studies among axSpA patients [7, 9, 10]. Additionally, we found a slightly higher drug discontinuation probability for IL-17i compared to TNFi. The explanation for that might be that the IL-17i treated patients in our cohort had seen more lines of treatment than the TNFi-treated patients. A study of secukinumab retention in axSpA patients revealed that drug survival was better when secukinumab was administered as first-line therapy rather than as a second- or third-line agent [8]. But when used as first-line bDMARD, drug survival of secukinumab did not differ from adalimumab or other TNFi [3, 9]. On the other hand, an observational cohort study of the 5 Nordish registries with almost 11 000 treatment courses indicated that drug retention of secukinumab might be inferior to adalimumab in the third line or higher [9].

Real-world data on the drug persistence of JAKi in axSpA are still not widely available. We only discovered one recent published study from the Spanish BIOBADASER registry with a similar sample size [11]. Their reported retention rate for all JAKi (1-year survival rate of 82.9%) was much higher than our reported rate, but at year 2, their reported survival rate of 64.0% was quite similar to the rates observed in our cohort [11]. When interpreting this discrepancy in the 1-year drug survival, it should be noted that the BIOBADASER study only registered 62 treatment courses with JAKi in axSpA, which corresponds to only 3/4  of the number of cases in our cohort [11]. Their Kaplan Meier curve was correspondingly angularly shaped [11]. Similar to our cohort, JAKi were mainly used in the third line or later, so that the more favorable 1-year survival cannot be easily explained by JAKi usage in an earlier line of therapy [11]. Our data might therefore be more accurate and depict the real-world drug survival more precisely due to the higher number of reported cases, but further studies will be needed to confirm our observations [11]. Interestingly, the probability for discontinuation of JAKi was significantly higher than for TNFi in adjusted Cox regression models in our cohort. For JAKi, this is surprising given studies revealing a strong patient preference for oral over subcutaneous therapies [27, 28]. The analysis from the Spanish BIOBADASER registry found no difference for drug discontinuation between TNFi and JAKi in their axSpA patients which might be interpreted with caution due to the smaller number of treatment courses as mentioned before [11]. On the other hand, the Danish DANBIO registry data and data published from the RHADAR database also showed significantly lower persistence rates for tofacitinib/JAKi compared with TNFi or IL-17i for PsA [3, 13]. When interpreting our drug survival rates of JAKi, it must be taken into account that JAKi-treated patients in our cohort had more previous treatments and higher disease activity parameters than TNFi-treated patients. A suitable interpretation of these findings might be that JAKi does not typically represent the first-line treatment of German axSpA outpatients and is a drug class reserved for more refractory forms of disease, which is in line with the findings reported for JAKi treated patients of the Spanish BIOBADASER register [11]. This would be consistent with observations on shorter drug survival times for patients having received a larger number of previous treatments [9, 11, 26]. Concomitant medication and the presence of osteoarthritis or population characteristics like age did not differ between the drug classes and, therefore, do not appear to explain the observed differences as in other registries [11]. To analyze whether the reason for drug discontinuation differed between the MoA we looked for the change in BASDAI and ASDAS values between treatment initiation and treatment switch as a surrogate for treatment response. The mean delta values exhibited only a mild negative delta and did not differ between the three MoA, indicating that the main reason for drug discontinuation for all MoA was non-response and not adverse events, which is in line with finding of other registries [9–11, 26].

It is also possible that the publication of the ORAL surveillance study data, which revealed an increased risk of cardiovascular events and cancer with tofacitinib, as well as the subsequent changes in the warnings and precautions section of the SmPCs of the JAKi may have prompted clinicians or patients to switch treatments [29, 30].

The findings of our study must be interpreted with caution due to several limiting factors. As a retrospective study based on data from a real-world clinical registry, drug survival was not evaluated prospectively, and groups were not matched or stratified. The RHADAR database is a registry with automatic data transfer from rheumatologic practices; these data are not monitored, which explains the considerable rate of missing values for some parameters. Not all available disease parameters were assessed in all patients, as it is common in routine clinical care, and reasons for discontinuation were not provided. For analysis purposes, specific agents within a drug class were aggregated, which may have affected the reported results. This effect might be more relevant for TNFi than other drug classes due to the greater number of agents in the TNFi drug class. Other studies have reported differences in drug persistence among TNFi inhibitors in patients with axSpA [3, 26]. Our analyses did not distinguish between AS (radiographic axSpA) and non-radiographic axSpA. Some studies, but not all, have found that patients with AS have a prolonged drug survival on bDMARDs compared with patients with non-radiographic axSpA [31–33].

Despite these limitations related to real-world studies, our findings provide unique and valuable insights into real-world drug retention rates of bDMARDs and JAKi in patients with axSpA during a time frame in which multiple agents with different MoA and modes of administration were available. High costs and the difficulty of performing study activities during routine outpatient care make it challenging to perform real-world drug survival studies. However, these studies are critical because they depict outcomes observed during routine care and may provide information to guide medical and economic decision-making in the healthcare sector.

Based on our data, we conclude that TNFi is still the preferred drug class for patients with axSpA who are initiating treatment with a new bDMARD or JAKi in the German outpatient sector. JAKi appear to be reserved for more severe or refractory forms of axSpA, and this preference could potentially be further intensified in the future by the findings of the ORAL surveillance study and the corresponding Pharmacolovigilance Risk Assessment Committee recommendations [29, 30]. Higher usage as first-line agents in patients with less severe or refractory disease might explain the longer drug survivals for TNFi compared with JAKi and IL-17i, but other factors, including effectiveness, may also play a role.

## Electronic supplementary material

Below is the link to the electronic supplementary material.

Supplementary Material 1

## Data Availability

The datasets generated during and/or analyzed during the current study are available from the corresponding author on reasonable request.

## References

[1] Maksymowych WP, Lambert RGW, Caplan L, van den Bosch FE, Østergaard M (2022) Improving the design of RCTs in non-radiographic axial spondyloarthritis. Nat Rev Rheumatol 18(8):481–489. 10.1038/s41584-022-00789-1Epub 2022 May 13. PMID: 35562426 35562426 10.1038/s41584-022-00789-1

[2] Gniadecki R, Bang B, Bryld LE, Iversen L, Lasthein S, Skov L (2015) Comparison of long-term drug survival and safety of biologic agents in patients with psoriasis vulgaris. Br J Dermatol 172(1):244–252. 10.1111/bjd.13343Epub 2014 Nov 30. PMID: 25132294 25132294 10.1111/bjd.13343

[3] Egeberg A, Rosenø NAL, Aagaard D, Lørup EH, Nielsen ML, Nymand L et al (2022) Drug survival of biologics and novel immunomodulators for rheumatoid arthritis, axial spondyloarthritis, psoriatic arthritis, and psoriasis - A nationwide cohort study from the DANBIO and DERMBIO registries. Semin Arthritis Rheum 53:151979 Epub 2022 Feb 9. PMID: 35183936 35183936 10.1016/j.semarthrit.2022.151979

[4] Kleinert S, Bartz-Bazzanella P, von der Decken C, Knitza J, Witte T, Fekete SP et al (2021) A Real-World Rheumatology Registry and Research Consortium: the German RheumaDatenRhePort (RHADAR) Registry. J Med Internet Res 23(5):e28164. 10.2196/28164PMID: 34014170; PMCID: PMC8176344 34014170 10.2196/28164 PMC8176344

[5] Baraliakos X, Kiltz U, Kononenko I, Ciurea A (2023) Treatment overview of axial spondyloarthritis in 2023. Best Pract Res Clin Rheumatol 37(3):101858. 10.1016/j.berh.2023.101858. Epub 2023 Sep 5. PMID: 37673758 10.1016/j.berh.2023.101858 37673758

[6] Danve A, Deodhar A (2022) Treatment of axial spondyloarthritis: an update. Nat Rev Rheumatol 18(4):205–216. 10.1038/s41584-022-00761-zEpub 2022 Mar 10. PMID: 35273385 35273385 10.1038/s41584-022-00761-z

[7] Yu CL, Yang CH, Chi CC (2020) Drug Survival of Biologics in Treating Ankylosing Spondylitis: A Systematic Review and Meta-analysis of Real-World Evidence. BioDrugs 34(5):669–679. 10.1007/s40259-020-00442-x. PMID: 32946076 10.1007/s40259-020-00442-x 32946076

[8] Dougados M, Lardy-Cléaud A, Desfleurs E, Claudepierre P, Goupille P, Ryussen-Witrand A et al (2024) Impact of the time of initiation and line of biologic therapy on the retention rate of secukinumab in axial spondyloarthritis (axSpA): data from the French multicentre retrospective FORSYA study. RMD Open 10(1):e003942. 10.1136/rmdopen-2023-003942PMID: 38428974; PMCID: PMC10910420 38428974 10.1136/rmdopen-2023-003942 PMC10910420

[9] Glintborg B, Lindström U, Giuseppe DD, Provan SA, Gudbjornsson B, Hetland ML et al (2022) One-year treatment outcomes of Secukinumab Versus Tumor necrosis factor inhibitors in Spondyloarthritis: results from five nordic Biologic registries Including more than 10,000 treatment courses. Arthritis Care Res (Hoboken) 74(5):748–758. 10.1002/acr.24523Epub 2022 Mar 8. PMID: 33253491 33253491 10.1002/acr.24523

[10] García-Dorta A, León-Suarez P, Peña S, Hernández-Díaz M, Rodríguez-Lozano C, González-Dávila E et al (2022) Association of Gender, diagnosis, and obesity with Retention Rate of Secukinumab in Spondyloarthropathies: results form a Multicenter Real-World Study. Front Med (Lausanne) 8:815881. 10.3389/fmed.2021.815881PMID: 35096907; PMCID: PMC8792854 35096907 10.3389/fmed.2021.815881 PMC8792854

[11] Hernández-Cruz B, Otero-Varela L, Freire-González M, Busquets-Pérez N, García González AJ, Moreno-Ramos M et al (2024) Janus kinase inhibitors and tumour necrosis factor inhibitors show a favourable safety profile and similar persistence in rheumatoid arthritis, psoriatic arthritis and spondyloarthritis: real-world data from the BIOBADASER registry. 10.1136/ard-2023-225271. Epub ahead of print. PMID: 38594056 Ann Rheum Dis ard-2023-225271 10.1136/ard-2023-225271 PMC11883748 38594056

[12] UCB (2023) https://www.ucb.com/stories-media/Press-Releases/article/UCB-Receives-New-European-Commission-Approvals-for-BIMZELXRVbimekizumab-for-the-Treatment-of-Psoriatic-Arthritis-and-Axial-Spondyloarthritis. Accessed June 23, 2024

[13] Strunz PP, Englbrecht M, Risser LM, Witte T, Froehlich M, Schmalzing M et al (2024) Drug survival superiority of tumor necrosis factor inhibitors and interleukin-17 inhibitors over Janus kinase inhibitors and interleukin-12/23 inhibitors in German psoriatic arthritis outpatients: retrospective analysis of the RHADAR database. Front Immunol 15:1395968. 10.3389/fimmu.2024.1395968PMID: 38846940; PMCID: PMC11153701 38846940 10.3389/fimmu.2024.1395968 PMC11153701

[14] Kiltz U, Braun J, DGRh, Becker A, Chenot DEGAM et al (2019) JF, Long version on the S3 guidelines for axial spondyloarthritis including Bechterew’s disease and early forms, Update 2019: Evidence-based guidelines of the German Society for Rheumatology (DGRh) and participating medical scientific specialist societies an. Z Rheumatol 78(Suppl 1):3–64. German. 10.1007/s00393-019-0670-3. PMID: 31784900 10.1007/s00393-019-0670-3 31784900

[15] Team R, Core R (2018) A language and environment for statistical computing. R Foundation for Statistical Computing, Vienna, Austria

[16] Team RStudio (2016) RStudio: Integrated Development for R. RStudio, Inc, Boston, MA

[17] Letarouilly JG, Salmon JH, Flipo RM (2021) Factors affecting persistence with biologic treatments in patients with rheumatoid arthritis: a systematic literature review. Expert Opin Drug Saf 20(9):1087–1094 Epub 2021 May 14. PMID: 33926364 33926364 10.1080/14740338.2021.1924146

[18] D’Amico ME, Silvagni E, Carrara G, Zanetti A, Govoni M, Scirè CA et al (2021) Role of comorbidities on therapeutic persistence of biological agents in rheumatoid arthritis: results from the RECord-linkage On Rheumatic Disease study on administrative healthcare databases. Scand J Rheumatol 50(5):333–342. doi: 10.1080/03009742.2020.1855365. Epub 2021 Mar 4. PMID: 33660559 10.1080/03009742.2020.1855365 33660559

[19] de Vries MK, Wolbink GJ, Stapel SO, de Vrieze H, van Denderen JC, Dijkmans BA et al (2007) Decreased clinical response to infliximab in ankylosing spondylitis is correlated with anti-infliximab formation. Ann Rheum Dis 66(9):1252–1254. 10.1136/ard.2007.072397Epub 2007 May 1. PMID: 17472991; PMCID: PMC1955152 17472991 10.1136/ard.2007.072397 PMC1955152

[20] Pérez-Guijo VC, Cravo AR, Castro Mdel C, Font P, Muñoz-Gomariz E, Collantes-Estevez E (2007) Increased efficacy of infliximab associated with methotrexate in ankylosing spondylitis. Joint Bone Spine 74(3):254-8. 10.1016/j.jbspin.2006.08.005. Epub 2007 Mar 1. PMID: 17387031 10.1016/j.jbspin.2006.08.005 17387031

[21] Arends S, Lebbink HR, Spoorenberg A, Bungener LB, Roozendaal C, van der Veer E et al (2010) The formation of autoantibodies and antibodies to TNF-α blocking agents in relation to clinical response in patients with ankylosing spondylitis. Clin Exp Rheumatol 28(5):661–668 Epub 2010 Oct 22. PMID: 20822711 20822711

[22] Albrecht K, Marschall U, Callhoff J (2021) Prescription of analgesics in patients with rheumatic diseases in Germany: A claims data analysis. Z Rheumatol 80(Suppl 2):68–75. doi: 10.1007/s00393-021-00971-y. Epub 2021 Apr 7. PMID: 33825975; PMCID: PMC8752520 10.1007/s00393-021-00971-y PMC8752520 33825975

[23] Krüger K, von Hinüber U, Meier F, Tian H, Böhm K, Jugl SM et al (2018) Ankylosing spondylitis causes high burden to patients and the healthcare system: results from a German claims database analysis. Rheumatol Int 38(11):2121–2131. 10.1007/s00296-018-4124-zEpub 2018 Aug 9. PMID: 30094685 30094685 10.1007/s00296-018-4124-z

[24] Haibel H, Redeker I, Zink A, Callhoff J, Marschall U et al (2019) Health care and disease burden in persons with axial spondyloarthritis in Germany. Z Rheumatol 78(9):865–874. German. 10.1007/s00393-019-0650-7. PMID: 31172266 10.1007/s00393-019-0650-7 31172266

[25] Redeker I, Callhoff J, Hoffmann F, Marschall U, Haibel H, Sieper J et al (2020) The prevalence and impact of comorbidities on patients with axial spondyloarthritis: results from a nationwide population-based study. Arthritis Res Ther 22(1):210. 10.1186/s13075-020-02301-0PMID: 32912264; PMCID: PMC7488243 32912264 10.1186/s13075-020-02301-0 PMC7488243

[26] Larid G, Baudens G, Tiemdjo-Djimaffo G, Coquerelle P, Goeb V, Guyot MH et al (2024) Retention rate of subcutaneous TNF inhibitors in axial spondyloarthritis in a multicentre study from the RIC-FRANCE network. Sci Rep 14(1):1374. 10.1038/s41598-024-52016-4PMID: 38228719; PMCID: PMC10791989 38228719 10.1038/s41598-024-52016-4 PMC10791989

[27] Sumpton D, Kelly A, Craig JC, Hassett G, Kane B, Oliffe M et al (2022) Preferences for Biologic treatment in patients with psoriatic arthritis: a Discrete Choice Experiment. Arthritis Care Res (Hoboken) 74(8):1234–1243. 10.1002/acr.24782Epub 2022 Jun 1. PMID: 34514744 34514744 10.1002/acr.24782

[28] Aletaha D, Husni ME, Merola JF, Ranza R, Bertheussen H, Lippe R et al (2020) Treatment Mode preferences in Psoriatic Arthritis: a qualitative Multi-country Study. Patient Prefer Adherence 14:949–961 PMID: 32606613; PMCID: PMC7293411 32606613 10.2147/PPA.S242336 PMC7293411

[29] Ytterberg SR, Bhatt DL, Mikuls TR, Koch GG, Fleischmann R, Rivas JL et al (2022) Cardiovascular and Cancer Risk with Tofacitinib in Rheumatoid Arthritis. N Engl J Med 386(4):316–326. 10.1056/NEJMoa2109927. PMID: 35081280 10.1056/NEJMoa2109927 35081280

[30] EMA (2023) https://www.ema.europa.eu/en/news/meeting-highlights-pharmacovigilance-risk-assessment-committee-prac-9-12-january-2023. Accessed 28 January 2024

[31] Ngo MD, Zummer M, Andersen KM, Richard N (2022) First Biologic Drug Persistence in Patients With Ankylosing Spondylitis and Nonradiographic Axial Spondyloarthritis: A Real-World Canadian Physicians’ Experience. J Clin Rheumatol 28(1):e210-e216. 10.1097/RHU.0000000000001693. PMID: 33394832 10.1097/RHU.0000000000001693 33394832

[32] Lopalco G, Venerito V, Cantarini L, Emmi G, Salaffi F, Di Carlo M et al (2019) Different drug survival of first line tumour necrosis factor inhibitors in radiographic and non-radiographic axial spondyloarthritis: a multicentre retrospective survey. Clin Exp Rheumatol 37(5):762–767 Epub 2019 Apr 16. PMID: 31025925 31025925

[33] Michelena X, Zhao SS, Dubash S, Dean LE, Jones GT, Marzo-Ortega H (2021) Similar biologic drug response regardless of radiographic status in axial spondyloarthritis: data from the British Society for Rheumatology Biologics Register in Ankylosing Spondylitis registry. Rheumatology (Oxford) 60(12):5795–5800. 10.1093/rheumatology/keab070PMID: 33502476; PMCID: PMC8645273 33502476 10.1093/rheumatology/keab070 PMC8645273

