# Supplementary material for: Analysis of the shorter drug survival times for Janus kinase inhibitors and interleukin-17 inhibitors compared with tumor necrosis factor inhibitors in a real-world cohort of axial spondyloarthritis patients - a retrospective analysis from the RHADAR network
Source: Rheumatol Int. 2024 Aug 13;44(10):2057–66. doi: 10.1007/s00296-024-05671-9 (PMC11392998; doi:10.1007/s00296-024-05671-9)
Supplement: Supplementary file 1 — Supplementary Material 1 [file 296_2024_5671_MOESM1_ESM.docx]

**Supplementary File to** **“Analysis of the shorter drug survival times for Janus kinase inhibitors and interleukin-17 inhibitors compared with tumor necrosis factor inhibitors in a real-world cohort of axial spondyloarthritis patients - a retrospective analysis from the RHADAR network” ​**

**Authors:** Patrick-Pascal Strunz MD^1^, Matthias Englbrecht PhD^2^, Linus Maximilian Risser MD^3^, Torsten Witte MD^3^, Matthias Froehlich MD^1^, Marc Schmalzing MD^1^, Michael Gernert MD^1^, Astrid Schmieder MD^4^, Peter Bartz-Bazzanella MD^5, 6^, Cay von der Decken MD^5, 6, 11^, Kirsten Karberg MD^7^, Georg Gauler MD^8^, Patrick Wurth MD^8^, Susanna Späthling-Mestekemper MD^9^, Christoph Kuhn MD ^10^, Wolfgang Vorbrüggen MD^11^, Johannes Heck^12^, Martin Welcker MD^11, 13^ and Stefan Kleinert MD^1, 14^

**Affiliations:** ^1^ University Hospital of Wuerzburg, Departement of Medicine II, Rheumatology/Clinical Immunology, Würzburg, Germany; ^2^ Freelance Healthcare Data Scientist, Greven, Germany; ^3^ Medical School Hannover, Department of Rheumatology and Immunology, Hannover, Germany; ^4^ Clinic for Dermatology, Venereology and Allergology, University Hospital Wuerzburg, Germany; ^5^ Klinik für Internistische Rheumatologie, Rhein-Maas-Klinikum, Würselen, Germany; ^6^ Medizinisches Versorgungszentrum, Stolberg, Germany; ^7^ Rheumatologisches Versorgungszentrum Steglitz, Berlin, Germany; ^8^ Rheumatology Practice, Osnabrück, Germany; ^9^ Rheumapraxis München¸ Germany; ^10^ Praxis für Rheumatologie, Karlsruhe, Germany; ^11^ Verein zur Förderung der Rheumatologie e.V., Würselen, Germany; ^12^ Hannover Medical School, Institute for Clinical Pharmacology, Hannover, Germany; ^13^ Medizinisches Versorgungszentrum für Rheumatologie Dr. M. Welcker GmbH, Planegg, Germany; ^14^ Praxisgemeinschaft Rheumatologie-Nephrologie, Erlangen, Germany.

**Supplemental section S1. Characteristics of the subpopulations stratified for the MoA**

| Patient characteristics of the IL-17i-subpopulation | | | | | | | | | | |
| --- | --- | --- | --- | --- | --- | --- | --- | --- | --- | --- |
|  | **Valid (n)** | **Valid (%)** | **Mean** | **95%CI (lower)** | **95%CI (upper)** | **SD** | **SEM** | **Median** | **25% Quantile** | **75% Quantile** |
| Gender (male) | 105 | 55.3 | NA | NA | NA | NA | NA | NA | NA | NA |
| Gender (female) | 85 | 44.7 | NA | NA | NA | NA | NA | NA | NA | NA |
| Age | 190 | 100.0 | 47.0 | 45.3 | 48.8 | 12.5 | 0.9 | 47.0 | 38.0 | 56.8 |
| Disease duration (years) | 160 | 84.2 | 12.7 | 10.9 | 14.4 | 11.4 | 0.9 | 8.0 | 4.0 | 20.2 |
| BASDAI | 127 | 66.8 | 5.3 | 4.9 | 5.6 | 2.1 | 0.2 | 5.2 | 3.9 | 7.0 |
| BASFI | 91 | 47.9 | 4.6 | 4.1 | 5.2 | 2.7 | 0.3 | 4.7 | 2.3 | 6.9 |
| ASDAS | 70 | 36.8 | 2.8 | 2.6 | 3.0 | 0.9 | 0.1 | 2.7 | 2.2 | 3.3 |
| ESR (mm/h) | 102 | 53.7 | 18.3 | 15.1 | 21.6 | 16.7 | 1.7 | 15.0 | 8.0 | 23.5 |
| CRP (mg/dl) | 130 | 68.4 | 1.1 | 0.5 | 1.8 | 3.9 | 0.3 | 0.3 | 0.2 | 0.6 |
| Pain (0-100) | 64 | 33.7 | 54.8 | 48.7 | 61.0 | 25.1 | 3.1 | 60.0 | 34.8 | 75.2 |
| Morning stiffness (min) | 57 | 30.0 | 56.6 | 44.2 | 69.0 | 47.7 | 6.3 | 60.0 | 30.0 | 66.0 |
| **Patient characteristics of the JAKi-subpopulation** | | | | | | | | | | |
|  | **Valid (n)** | **Valid (%)** | **Mean** | **95%CI (lower)** | **95%CI (upper)** | **SD** | **SEM** | **Median** | **25% Quantile** | **75% Quantile** |
| Gender (male) | 35 | 44.9 | NA | NA | NA | NA | NA | NA | NA | NA |
| Gender (female) | 43 | 55.1 | NA | NA | NA | NA | NA | NA | NA | NA |
| Age | 78 | 100.0 | 50.0 | 47.0 | 53.0 | 13.5 | 1.5 | 52.5 | 40.2 | 58.0 |
| Disease duration (years) | 64 | 82.1 | 12.7 | 10.1 | 15.2 | 10.5 | 1.3 | 9.5 | 5.8 | 17.0 |
| BASDAI | 56 | 71.8 | 5.2 | 4.6 | 5.7 | 2.2 | 0.3 | 5.0 | 3.9 | 6.5 |
| BASFI | 43 | 55.1 | 4.7 | 3.9 | 5.6 | 2.9 | 0.4 | 4.0 | 2.3 | 7.2 |
| ASDAS | 40 | 51.3 | 3.0 | 2.7 | 3.3 | 0.8 | 0.1 | 2.9 | 2.5 | 3.3 |
| ESR (mm/h) | 48 | 61.5 | 19.9 | 14.9 | 24.9 | 17.6 | 2.5 | 12.5 | 10.0 | 23.0 |
| CRP (mg/dl) | 57 | 73.1 | 0.8 | 0.4 | 1.2 | 1.5 | 0.2 | 0.3 | 0.3 | 0.5 |
| Pain (0-100) | 31 | 39.7 | 55.3 | 45.4 | 65.3 | 28.2 | 5.1 | 60.0 | 45.5 | 80.0 |
| Morning stiffness (min) | 28 | 35.9 | 166.3 | 31.1 | 301.5 | 365.0 | 69.0 | 53.0 | 30.0 | 99.0 |

| Patient characteristics of the TNFi-subpopulation | | | | | | | | | | |
| --- | --- | --- | --- | --- | --- | --- | --- | --- | --- | --- |
|  | **Valid (n)** | **Valid (%)** | **Mean** | **95%CI (lower)** | **95%CI (upper)** | **SD** | **SEM** | **Median** | **25% Quantile** | **75% Quantile** |
| Gender (male) | 528 | 55.3 | NA | NA | NA | NA | NA | NA | NA | NA |
| Gender (female) | 426 | 44.7 | NA | NA | NA | NA | NA | NA | NA | NA |
| Age | 954 | 100.0 | 46.7 | 45.8 | 47.6 | 13.6 | 0.4 | 47.0 | 37.0 | 57.0 |
| Disease duration (years) | 823 | 86.3 | 12.6 | 11.8 | 13.4 | 11.3 | 0.4 | 9.0 | 4.0 | 19.0 |
| BASDAI | 624 | 65.4 | 4.1 | 3.9 | 4.2 | 2.2 | 0.1 | 4.1 | 2.3 | 5.7 |
| BASFI | 461 | 48.3 | 3.3 | 3.1 | 3.6 | 2.6 | 0.1 | 2.8 | 1.1 | 5.2 |
| ASDAS | 311 | 32.6 | 2.4 | 2.3 | 2.5 | 0.9 | 0.1 | 2.4 | 1.7 | 3.0 |
| ESR (mm/h) | 487 | 51.0 | 18.6 | 17.0 | 20.1 | 17.7 | 0.8 | 12.0 | 7.0 | 24.0 |
| CRP (mg/dl) | 589 | 61.7 | 0.6 | 0.5 | 0.8 | 1.4 | 0.1 | 0.3 | 0.1 | 0.6 |
| Pain (0-100) | 273 | 28.6 | 37.0 | 33.8 | 40.2 | 27.1 | 1.6 | 36.0 | 10.0 | 60.0 |
| Morning stiffness (min) | 257 | 26.9 | 63.0 | 42.9 | 83.0 | 163.8 | 10.2 | 30.0 | 14.0 | 60.0 |

**Supplemental section S2. Concomitant Therapies**

1. TNFi vs. IL-17i

|  | **Monotherapy** | **Combined therapy** | **Total** |
| --- | --- | --- | --- |
| **TNFi** | 849 | 105 | 954 |
| **IL-17i** | 173 | 17 | 190 |
| **Total** | 1022 | 122 | 1144 |

Χ², df: 0.7050, 1; OR: 0.7945 95% CI: 0.4640 to 1.361 p= 0.4011

1. TNFi vs. JAKi

|  | **Monotherapy** | **Combined therapy** | **Total** |
| --- | --- | --- | --- |
| **TNFi** | 849 | 105 | 954 |
| **JAKi** | 65 | 13 | 78 |
| **Total** | 914 | 118 | 1032 |

Χ², df: 2.281, 1; OR: 1.617 95% CI: 0.8620 to 3.034 p= 0.1309

1. JAKi vs. IL-17i

|  | **Monotherapy** | **Combined therapy** | **Total** |
| --- | --- | --- | --- |
| **JAKi** | 65 | 13 | 78 |
| **IL-17i** | 173 | 17 | 190 |
| **Total** | 238 | 30 | 268 |

Χ², df: 3.3150, 1; OR: 0.4913 95% CI: 0.2260 to 1.068 p= 0.0687

**3. Osteoarthritis**

1. TNFi vs. IL-17i

|  | **Osteoarthritis** | **No Osteoarthritis** | **Total** |
| --- | --- | --- | --- |
| **TNFi** | 169 | 785 | 954 |
| **IL-17i** | 45 | 145 | 190 |
| **Total** | 214 | 930 | 1144 |

Χ², df: 3.713, 1; OR: 0.6937 95% CI: 0.4775 to 1.008 p= 0.0540

1. TNFi vs. JAKi

|  | **Osteoarthritis** | **No Osteoarthritis** | **Total** |
| --- | --- | --- | --- |
| **TNFi** | 169 | 785 | 954 |
| **JAKi** | 18 | 60 | 78 |
| **Total** | 187 | 845 | 1032 |

Χ², df: 1.397, 1; OR: 0.7176 95% CI: 0.4130 to 1.247 p= 0.2372

1. JAKi vs. IL-17i

|  | **Osteoarthritis** | **No Osteoarthritis** | **Total** |
| --- | --- | --- | --- |
| **IL-17i** | 45 | 145 | 190 |
| **JAKi** | 18 | 60 | 78 |
| **Total** | 63 | 205 | 268 |

Χ², df: 0.01134, 1; OR: 1.034 95% CI: 0.5542 to 1.931 p= 0.9152

Supplemental Section S3: Cox regression

1. Cox-Regression - reference: TNF

|  | Coefficient | Hazard Ratio (HR) | HR (lower 95%CI) | HR (upper 95%CI) | SE coefficient | z | p-value |
| --- | --- | --- | --- | --- | --- | --- | --- |
| IL-17 | 0.36 | 1.43 | 1.02 | 2.01 | 0.17 | 2.08 | 0.037 |
| JAK | 0.65 | 1.91 | 1.22 | 2.99 | 0.23 | 2.82 | 0.005 |
| Gender (female) | 0.68 | 1.97 | 1.50 | 2.59 | 0.14 | 4.91 | 0.000 |
| Age | 0.00 | 1.00 | 0.99 | 1.01 | 0.01 | -0.76 | 0.448 |
| Disease duration | -0.02 | 0.98 | 0.97 | 0.99 | 0.01 | -2.69 | 0.007 |

2. Cox-Regression - reference: IL-17

|  | Coefficient | Hazard Ratio (HR) | HR (lower 95%CI) | HR (upper 95%CI) | SE coefficient | z | p-value |
| --- | --- | --- | --- | --- | --- | --- | --- |
| JAK | 0.29 | 1.33 | 0.79 | 2.24 | 0.26 | 1.08 | 0.281 |
| TNF | -0.36 | 0.70 | 0.50 | 0.98 | 0.17 | -2.08 | 0.037 |
| Gender (female) | 0.68 | 1.97 | 1.50 | 2.59 | 0.14 | 4.91 | 0.000 |
| Age | 0.00 | 1.00 | 0.99 | 1.01 | 0.01 | -0.76 | 0.448 |
| Disease duration | -0.02 | 0.98 | 0.97 | 0.99 | 0.01 | -2.69 | 0.007 |

3. Cox-Regression - reference: JAK

|  | Coefficient | Hazard Ratio (HR) | HR (lower 95%CI) | HR (upper 95%CI) | SE coefficient | z | p-value |
| --- | --- | --- | --- | --- | --- | --- | --- |
| IL-17 | -0.29 | 0.75 | 0.45 | 1.26 | 0.26 | -1.08 | 0.281 |
| TNF | -0.65 | 0.52 | 0.33 | 0.82 | 0.23 | -2.82 | 0.005 |
| Gender (female) | 0.68 | 1.97 | 1.50 | 2.59 | 0.14 | 4.91 | 0.000 |
| Age | 0.00 | 1.00 | 0.99 | 1.01 | 0.01 | -0.76 | 0.448 |
| Disease duration | -0.02 | 0.98 | 0.97 | 0.99 | 0.01 | -2.69 | 0.007 |
